# Plant Behavior: Theoretical and Technological Advances

**DOI:** 10.1016/j.copsyc.2025.102026

**Published:** 2025-08

**Authors:** Bianchi Margherita, Avesani Sara, Bonato Bianca, Dadda Marco, Guerra Silvia, Ravazzolo Laura, Simonetti Valentina, Castiello Umberto

**Affiliations:** Department of General Psychology, University of Padua; DAFNAE-Department of Agronomy, Food, Natural Resources, Animals and Environment, University of Padua; Department of General Psychology, University of Padua

## Abstract

The widespread disregard for plant behavior is gradually being overcome through more inclusive theoretical approaches and the development of appropriate and advanced technologies.

In this paper we review scientific evidence on recent contributions to the study of plants, such as movement and communication as well as potential forms of attention. Some of the most recent contributions to the study of plant abilities come from comparative studies on biocommunication and research on the accuracy of plants in responding to different environmental stimuli through electrophysiological and kinematical analyses in different context (e.g., individual and social). Further, an underexplored area that warrants further investigation is plants’ multisensory perception and its potential link to multimodal communication capabilities.

Research into this set of abilities could help to clarify the degree of behavioral flexibility in sessile organisms without a nervous system and enhance discussions on interactive behavior as expressed in nature. This, in turn, will help to bridge the gap between studies on animal organisms and the rest of the living world.

## Introduction

In recent years, attention towards the plant kingdom has increased, extending beyond the traditional levels of study (e.g., taxonomy, physiology, ecology). The new entry in the field is the analysis of plants’ cognitive state [1*, 2, 3, 4]. Growing evidence suggests that plants possess abilities associated with decision-making, communication, memory and learning, anticipatory behavior, and more. This aspect is very interesting to explore because plants are rooted organisms without a nervous system (resulting in different structure, functional organization and ecological interactions).

Several reasons have contributed to the long-standing neglect of plant (cognitive) behavior. Chief among them is that we were neither theoretically nor methodologically equipped to fully understand how these processes unfold in plants. What has changed, and continues to change, is the development of more inclusive cognitive theories and technologies tailored to better understand the ability of plants to interact with each other and with other organisms.

In first instance, the emergence of theoretical frameworks such as the 4E cognition program [5, 6], systemic and relational approaches [7] and predictive models [8, 9] has certainly contributed. At a general level, these approaches have enabled us to view plants as organisms capable of deploying various behavioral strategies to solve survival challenges in changing environments. Special attention was paid to the specific body organization of plants, their interspecies and interregnum relationships with other organisms, and their ability to store information and anticipate environmental changes. In second instance, advancements in instrumentation and techniques, such as those for detecting chemical-electric signaling dynamics, analyzing volatile organic compounds (VOCs) for chemical communication, and studying plant movement through 3D analysis, have marked a turning point. At this stage, a natural question arises: to what extent have these factors influenced our insights into plant behavior? A realistic answer lies in the interrelation between the two levels (theoretical and practical developments) and the gradual acceptance and familiarization with this type of inquiry by researchers in different research domains.

To historically illustrate the close interplay between theoretical and practical advances, it is relevant to consider how shifts in mindset and technological progress have catalyzed pivotal moments in plant behavioral research. Pioneering analyses of electrical signaling in plants by Sir Jagadish Chandra Bose (1858-1937), early studies on the motor capabilities of insectivorous and climbing plants by Charles R. Darwin (1809-1882) and Francis Darwin (1848-1925), and time-lapse observations by Wilhelm Friedrich Pfeffer (1845-1920) serve as prime examples. Further, notable contributions include the discovery of plant hormones, beginning with Frits Went’s identification of auxin in the 1920s, which revolutionized our understanding of plant growth and development, and the breakthroughs in molecular biology in the late 20th century that unraveled signaling pathways and gene regulation in response to environmental stimuli. All this underscores that studies on plant activity didn’t emerge out of nowhere [10]; rather, they have taken time to gain recognition.

## Contemporary Research on Plant Signaling and Communication

One of the goals of contemporary research into plant capabilities is to deepen our understanding of the “chemical alphabet of plants” and how the type, quantity, and context of a released substance influence the information being transmitted [11*]. These studies aim to position the various levels of plant signaling (primarily, but not exclusively, chemical) within the broader field of “biocommunication”, which examines the diverse communicative abilities of various organisms across the evolutionary tree, moving beyond research focused solely on human linguistic communication [12**, 13].

Historically, at least since it was observed that some herbivorous insects found tree leaves less attractive when nearby trees had already been attacked by other insects, it has been suggested, and later confirmed, that damaged plants release volatile signals, consequently preparing nearby healthy plants for defense [14]. Although it is likely that this is not the equivalent of intentional prosocial behavior in aneural organisms [15], certainly this type of signaling promotes the collective well-being of adjacent plants.

Nowadays, we know that several volatile organic compounds (VOCs) are spontaneously biosynthesized and released by healthy plants even in the absence of induced stress [16]. Plants primarily use VOCs for reproductive purposes (attracting pollinators), defense (against herbivores and pathogens), and to signal stressors [17]. The chemical profiles, concentration, and timing of VOC release also serve as indicators for identifying nearby plants and their health status. Thus, VOCs appear to act as key signals through which plants communicate and interact with their environment [18].

From a technological standpoint, real-time monitoring of VOCs is of particular interest. Greater understanding of plant VOC biochemistry and ecology has led to the development of a variety of systems for their collection and analysis. The choice of the system for a given experiment depends on the specific biological question and the plant material under investigation [19]. Conventional methods often involve destructive approaches, such as sampling a portion of plant tissue followed by extraction and/or solvent distillation. Analyzing living systems provides more representative VOC emissions and more reliable data [20].

## Perceiving and Attending: The Electrome

A cutting-edge area of research into plant abilities focuses on the potential presence of forms of “attention.” These studies stem from the observation of plants’ behavioral flexibility in response to various stimuli and interaction contexts [21–23].

In general, attentional capacities are closely linked to the ability to exhibit goal-directed behavior, allowing an organism to orient its responses appropriately within a given environment. This involves distinguishing relevant elements in a context and selectively engaging with specific aspects while ignoring others [24, 25**].

Research on potential attentional processes in plants has been connected to the study of electrical signaling [26–28], leading to the development of electrodes capable of monitoring changes in electrical activity within plant tissues [29]. Specifically, Parise et al. [30*, 31] have proposed the use of electrophysiological analysis techniques to investigate changes in the overall bioelectrical activity of plants, their “electrome” (the totality of electrical activity in an organism or its parts over a given period, in response to various ecological stimuli, both artificial and natural). The change in electrical signaling could be linked to important processes such as foraging. One example involves measuring variations in the electrome of parasitic plant *C. racemosa* when exposed to bean (i.e., suitable host) or wheat (i.e., unsuitable host). The findings revealed that *Cuscuta*’*s* electrome exhibited a higher energy power and organization in the presence of a potential nutrient source (suitable host). This case is particularly intriguing because the task, which leads to changes in the electrome, requires a coordinated signaling effort from the entire plant: something that is not typically observed in modular organisms composed of semi-autonomous units [32].

Overall, investigating forms of “attention” in plants could have significant potential for understanding how sessile, aneural organisms can enhance the accuracy of their responses to address survival challenges. In this context, attention could be understood as a selection process driving goal-directed behavior [33].

## Anticipating the Course of an Action: Plants Movement Kinematics

Concerning the study of plant movement, it is now well established that plants are capable of various types of movement, despite their sessile nature. The ability of plants to move, crucial for exploring the environment, seeking light with aerial parts, and nutrients with roots (foraging), is essential for their survival [34].

Since the introduction of time-lapse photography, plant movements have become perceivable to us [35]. The aim of introducing these techniques was not to “animalize” plant behavior but to make it visible and more understandable to humans, who are sensitive to a different temporal scale. It’s worth noting that the opposite practice, slowing down animal movements, is commonly used to study the behavior of many animal species in nature [6].

Charles Darwin and his son Francis were among the first to detect and observe plant movements, particularly in climbing plants. The Darwins [36, 37] discovered that plants are capable of rejecting unsuitable supports (e.g., those too smooth or too large) and “planning” their movements based on the goals they need to achieve. It appears that plants can anticipate their movements before reaching a support. This insight sheds light on how plants respond to different stimuli in various contexts, enhancing our understanding of their ecological adaptations.

Recently, three-dimensional kinematic analysis of circumnutation variation – the elliptical movement of the apical sections of young growing plants – has revealed plants’ ability to adjust the opening of the tendrils (i.e., modified leaves used to grasp potential supports like a wooden stick) and to regulate the speed of approach to supports [38, 39]. Comparisons with the literature on motor control in animals indicate that plant movements follow the *speed-accuracy* tradeoff [40, 41] and the principle of isochrony [42]. Additionally, the production of submovements has been observed, depending on the characteristics of the support [41] and the context, suggesting that plants exert control over the precision of their movements, both in individual and social interactions involving the presence of other plants [43]. All these groundbreaking studies offer fascinating insights into the “action” capabilities of plants and may reveal aspects of motor intentionality in organisms without a brain [44*].

## Further Developments in Understanding Plant Behavior: Multisensoriality and Multimodality

An interesting area for future research, which remains largely unexplored, is the potential ability of plants to engage in multisensory perception and possibly multimodal communication to organize behavior.

We already know that plants possess sensory capabilities analogous to those of animals, though differently organized and distributed. Indeed, plant behavior is regulated by complex signaling pathways that begin with the perception of biotic (i.e., other living things) and abiotic (i.e., nonliving chemical and physical elements) environmental stimuli [45]. Many receptors and mechanosensitive channels are involved in the sensory capabilities of plants. Given that plants have multiple sensory and communicative abilities, it remains unclear how interconnected these channels are. Broadly speaking, one might ask what the purpose of multisensory perception is for plants. Having multiple senses and forms of signaling seems advantageous, as it could broaden the range of perceivable signals [46], particularly in noisy environments where signals from a single modality may become less effective, increasing the risk of signal loss [47*]. Thus, multisensoriality, in plants as in other organisms, may provide a survival advantage by enhancing communication in variable contexts.

Regarding the concept of multimodality, it is important to clarify that it is related to, but distinct from, multisensoriality. “Multimodal” refers to different modes of communication, whereas “multisensory” refers to the use of multiple senses. Moreover, it is essential to distinguish between “multicomponent” and “multimodal” communication. The former refers to multiple signal elements within a single communication modality [48], while the latter involves multiple signal elements across different sensory modalities [49].

From an ecological perspective, signal effectiveness is enhanced when communication occurs simultaneously through multiple modalities. Multimodal signaling represents a form of phenotypic plasticity, where one sensory channel compensates for another that is weak or underdeveloped [50]. Multimodal signals appear to be more effective than unimodal ones in transmitting information, increasing signal salience [51], improving memory retention of signals, and clarifying the sender’s intent [52]. In species with complex behaviors, multimodal communication can lead to the emergence of new meanings [49] and highlight cognitive capabilities in the ability to switch between modalities [53]. All these aspects concerned with a potential communication enhancement warrant further exploration in plant interactions, to determine what is specific and unique to plants and what is shared with the animal kingdom.

In multimodal signaling there are anatomical and physiological constraints imposed by the various sensory channels [54**]. Different signals have distinct characteristics, such as varying transmission distances and persistence. The “active space” of action of a signal – how far it can travel or be perceived – differs by sensory channel. For instance, chemical signals travel more slowly and are significantly affected by spatial transmission factors, such as temperature, wind, and humidity [55]. Given these differences in sensory channels and communication modes, the issue of signal integration arises. Signals that are closer in time and space are more likely to be captured and integrated [46].

Since behavior depends on the integration of information across different spatial and temporal scales, a future goal will be to explore the trade-offs between integration mechanisms and non-causal organization of information, even in non-neural organisms like plants [56, 57, 33].

More specifically, aspects involving multisensoriality and multimodality communication are beginning to be studied in plants, particularly in their interactions with pollinators. One example is the simultaneous presentation of information across multiple sensory modalities in flowers, which can be detected by bumblebees. While earlier research focused on visual and olfactory information, more recent attention has shifted to additional cues such as humidity, electric potential, surface texture, and flower temperature, with findings suggesting that multimodal signals enhance pollinator performance in locating pollen [47*].

Given our focus, these studies on multisensoriality and multimodality are crucial for better understanding plant behavior, including their ability to anticipate and plan movements, which is evident in their goal-directed behaviors.

From research on human and other animal abilities, such as studies on reach-to-grasp movements, we know that multisensory perception is involved in testing the cognitive architecture of goal-directed actions. Understanding these behaviors is key to grasping how organisms interact with their environment. Indeed, research has shown that how reach-to-grasp movements are planned and executed is influenced by information from multiple sensory modalities, such as vision, proprioception, hearing, taste, and smell [58].

Gaining a better understanding of how attention selection, multisensory integration, and individual and social intentionality shape actions has significant implications for understanding the planning and real-time control of movements aimed at survival and communication. Much remains to be discovered regarding the best theoretical approaches and the most efficient technologies (an almost unexplored territory) to elucidate aspects of multisensoriality and the potential presence of multimodal communication capabilities in plants.

## Conclusions and Future Perspectives

A future goal is to provide a more integrated understanding of plants’ individual capabilities to better grasp, from an interactional perspective, how plants perceive and respond with a certain level of accuracy to their environment. To clarify these aspects, we might ask whether plants communicate solely through “multicomponent” modes or if they truly engage in “multimodal” communication.

Another issue to explore is whether we can distinguish between “fixed” signals in plants, where a signal of one type, such as “visual”, is always linked to another type, like acoustic or chemical/olfactory, and “free” signals, where multiple signals do not necessarily occur together [59, 48]. Do plants exhibit combinations of fixed or free signals, or both? How much variability is possible? A closely related question is whether plants display sensory dominance in certain behaviors or interaction contexts, and whether, in terms of signaling, they can switch multimodally between sensory channels when there is too much noise that could cause signal disruption. Another goal could be to explore and quantify the changes in sensory and signaling abilities during plant development, and to understand which ecological contexts and the presence of which organisms are most relevant during the different life stages of a plant species [60, 49].

Moreover, from a practical standpoint, understanding how different sensory-perceptual channels interact, and the possibility of utilizing multiple forms of signaling simultaneously or switching between them, could also aid in predicting how plants respond to climatic changes that affect communication channels [54**, 61*, 62].

A deeper understanding of multisensory processing and potential multimodal communication in plants will help determine the extent of behavioral flexibility in plants, and concurrently bridging the gap between studies on human capabilities, those on other animals, and the rest of the living world. This will allow us to view plants – and the other organisms that seem distant in terms of structural and functional organization and ecological relationships – as less alien. The benefit will be that we can better interpret not only the ever-changing differences among ecosystems but also the diverse forms and level of biocommunication and behavior, thereby strengthening our connection with the entire natural community, redefining boundaries while highlighting both similarities and differences.

## Figures and Tables

**Figure 1:**
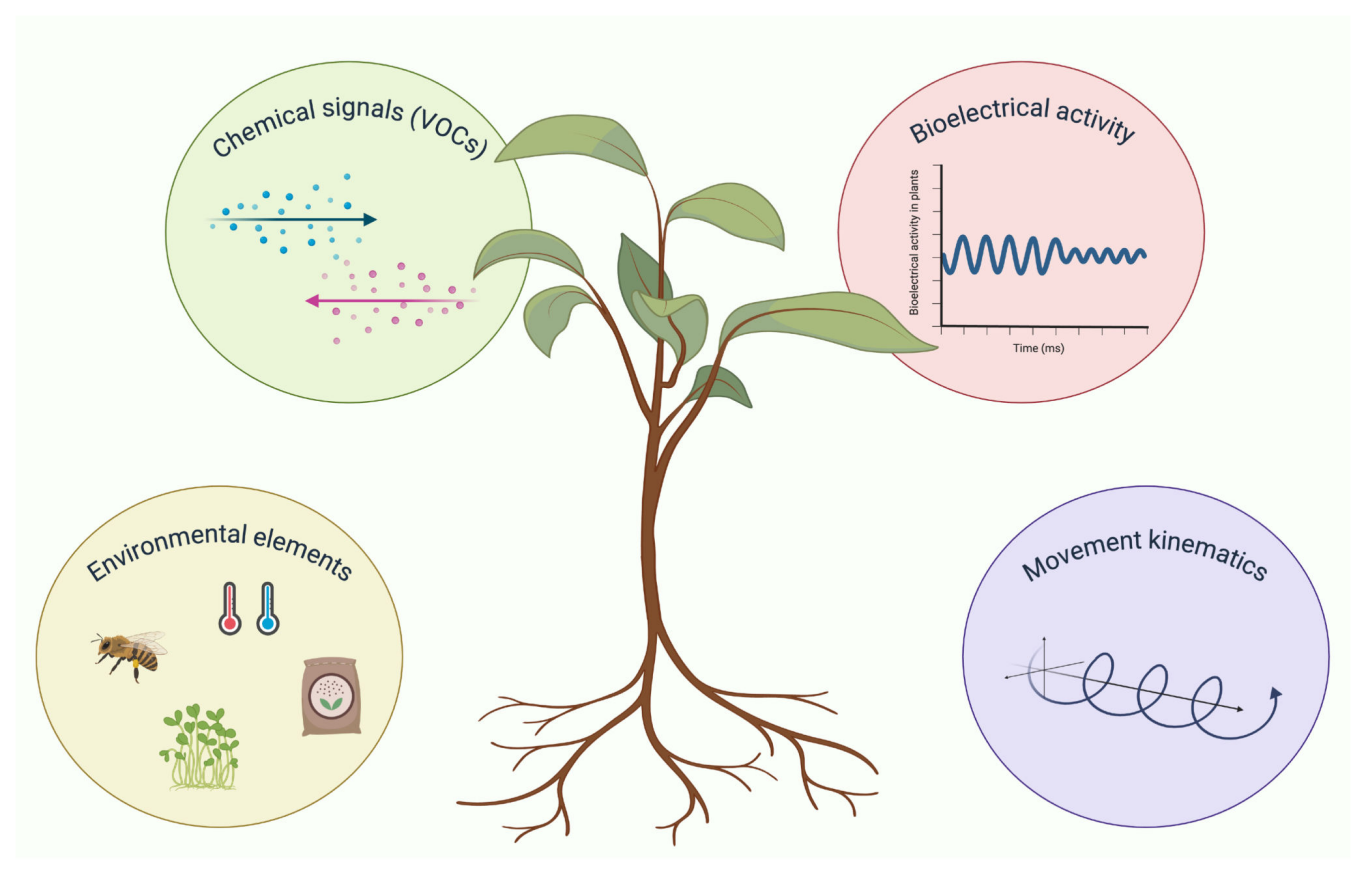
Plants’ Multilevel Perception and Communication Schematic representing the ways to examine behavioral flexibility in plants in the framework of cognition. The figure was created with Biorender.com.
